# Investigation of the adsorption capacity of the enterosorbent Enterosgel for a range of bacterial toxins, bile acids and pharmaceutical drugs

**DOI:** 10.1038/s41598-019-42176-z

**Published:** 2019-04-04

**Authors:** Carol A. Howell, Sergey V. Mikhalovsky, Elena N. Markaryan, Alexander V. Khovanov

**Affiliations:** 1 University of Brighton, School of Pharmacy & Biomolecular Sciences, Brighton, BN2 4GJ UK; 2 ANAMAD Ltd, Science Park Square, Brighton, BN1 9SB UK; 3 SD Asfendiyarov Kazakh National Medical University, Almaty, 050000 Kazakhstan; 4 Enteromed Ltd, London, W1W 7LT UK; 5 TNK SILMA LLC, Shipilovskaya, Moscow, Russia

## Abstract

Oral intestinal adsorbents (enterosorbents) are orally administered materials which pass through the gut where they bind (adsorb) various substances. The enterosorbent Enterosgel (Polymethylsiloxane polyhdrate) is recommended as a symptomatic treatment for acute diarrhoea and chronic diarrhoea associated with irritable bowel syndrome (IBS). Since 1980’s there have been many Enterosgel clinical trials, however, the detailed mechanism of Enterosgel action towards specific toxins and interaction with concomitantly administered medications has not been fully investigated. Our *in vitro* study assessed the adsorption capacity of Enterosgel for bacterial enterotoxins and endotoxin, bile acids and interaction with the pharmaceutical drugs; Cetirizine and Amitriptyline hydrochloride. Our data demonstrate the good adsorption capacity of Enterosgel for bacterial toxins associated with gastrointestinal infection, with a lower than the comparator charcoal Charcodote capacity for bile acids whose levels can be raised in IBS patients. Adsorption capacity for the two drugs varied but was significantly lower than Charcodote. These findings suggest that the mechanism of Enterosgel action in the treatment of gastrointestinal infection or IBS is adsorption of target molecules followed by removal from the body. This therapy offers a drug free approach to prevention and treatment of infectious and chronic non-infectious diseases, where intestinal flora and endotoxemia play a role.

## Introduction

The term “enterosorption” was first defined as the net gain of substances into the alimentary canal^1^ and in the context of a method of sorption therapy using orally administered sorbent materials or “enterosorbents”, was first used in the Soviet Union^2^. Enterosorbents are a group of materials which include activated carbons or charcoals, inorganic minerals, polymeric and silicon-containing resins. More recently they have been termed oral or intestinal adsorbents. The term enterosorption is used to describe the process in which an enterosorbent travels along the gastrointestinal tract during which time it can adsorb certain molecules, but itself is neither absorbed from the gut into the systemic circulation, nor metabolised and is thus excreted unchanged^3^. Charcoal has been used medically for many centuries, but it is only since the 1940s that its gastrointestinal adsorbent capacity has been demonstrated^4^. For acute poisoning activated charcoal use is the intervention of choice to eliminate the poison from the gastrointestinal tract (GIT) and prevent its absorption by the body^5^. Enterosorbents derived from calcium montmorillonite clays, such as NovaSil have been shown to adsorb aflatoxin B1 *in vitro* and^6^ be clinically effective in decreasing biomarkers of aflatoxin in the blood^7^. However, charcoal is limited to short-term use as they are potent non-selective adsorbents. Regular use of enterosorbents over a longer period to treat medical conditions associated with chronic intoxication where sequestration of gut of toxins produced by the body or from bacterial species could be beneficial to patients, require a different set of physicochemical characteristics than for acute poisoning treatment. These include a material that is safe to be used over the longer-term, which can still adsorb target molecules such as bacterial toxins but shows negligible interactions with medications and micronutrients.

Enterosgel is an enterosorbent with an excellent safety record for over 30 years in the CIS countries, where it has been used in newborns to the elderly. It is listed in the governmental guidelines to treat wide range of pathologies from acute intestinal infections to side effects of chemo- and radiotherapy. In Europe, Enterosgel is a class IIa medical device and recommended as a symptomatic treatment of acute diarrhoea and chronic diarrhoea associated with Irritable Bowel Syndrome (IBS). Recent clinical studies have confirmed that Enterosgel improves the outcome in children and adults with acute diarrhoea (gastroenteritis)^8–11^, chronic diarrhoea including IBS with diarrhoea (IBS-D)^12,13^ and therapy induced diarrhoea in cancer patients^14^. Enterosgel is an organosilicon compound, polymethylsiloxane polyhydrate (PMSPH) which is formed from methylsiloxane by polycondensation. It comprises microglobules which contain porous space filled with water^15,16^; these connect into larger particles (<250 µm in size) forming a hydrogel. The gel is amorphous and insoluble in water. Owing to its gelatinous nature, Enterosgel has a unique porous structure with a specific surface area of 150–250 m^2^/g dry weight^16^. It has pores in the microporous range (below 2 nm in diameter) but mostly wide mesopores and small macropores in the range from 2 nm up to 100 nm in diameter^17^. Enterosgel is composed of PMSPH and water in the ratio of 70/30 by weight, which has been found experimentally to have the highest pore volume ca. 1.5–1.6 cm^3^/g^18^.

Generally, enterosorbents are non-selective in their adsorption, however, Enterosgel is reported as having an unusual and unique adsorption profile, showing an increasing sorption capacity with the increase in the molecular weight of the solute^19^. This characteristic could be advantageous in limiting the unwanted adsorption of small molecules such as pharmaceutical drugs. Previous studies by Bardakhivska, *et al*.^20,21^, have shown that Enterosgel has lower adsorption activity towards certain anti-tuberculosis and anti-HIV preparations compared to activated charcoal.

Although Enterosgel is used in many clinical applications, the main mechanism of therapeutic action is still not fully understood; it is thought to be molecular adsorption of biological toxins from the gut. In this article we investigated Enterosgel’s mechanism of action, by examining *in vitro* adsorption capacity for bacterial toxins and bile acids, and interaction with the selected pharmaceutical drugs Cetirizine and Amitriptyline hydrochloride, assessing Enterosgel’s potential as a supportive therapy in complex treatment of conditions associated with the build-up of toxins in the GIT.

## Results

### Adsorption of bacterial enterotoxins and endotoxin by Enterosgel

**Figure 1:**
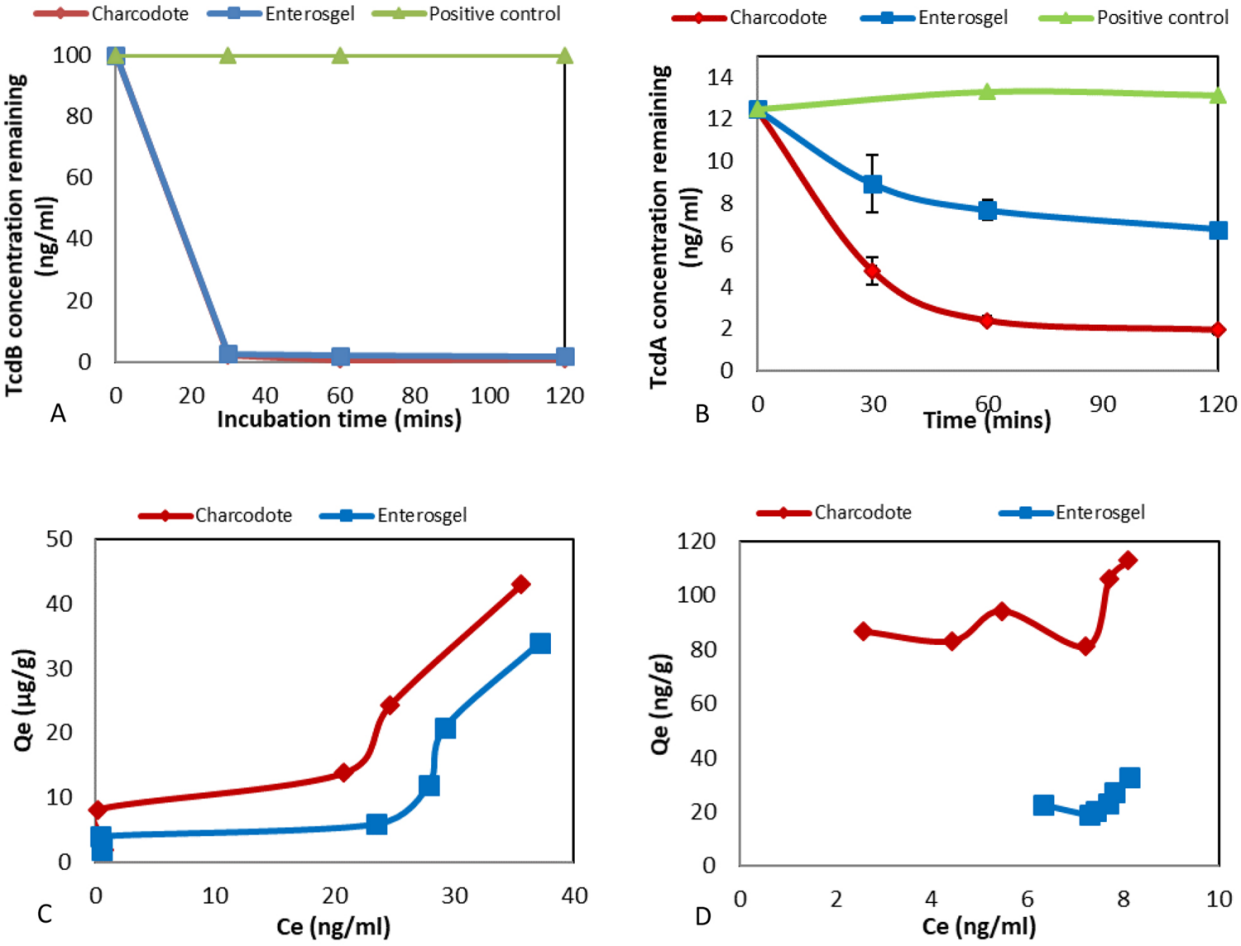
Adsorption kinetics for TcdB (**A**) and TcdA (**B**), showing remaining concentration over time for Enterosgel, Charcodote and positive control (no adsorbent) (mean ± sem). Equilibrium adsorption isotherm (Qe) of TcdB (**C**) and TcdA (**D**) against remaining concentration in solution (Ce) for Enterosgel and Charcodote.

The concentrations of Shiga Toxin II subunit B (Stx-2B) and *Clostridium difficile* Toxin A (TcdA) and Toxin B (TcdB) remaining after incubation with the adsorbents were determined using the equation of the standard calibration curve using a sigmoidal 4PL curve with R^2^ values of 0.9987, 0.9939 and 0.999 respectively. Kinetic adsorption results for TcdB (Fig. 1A) indicated that Enterosgel was able to adsorb most of the TcdB within the first 30 min of incubation similar to the activated carbon Charcodote (p = 0.98). In Fig. 1B, the results for TcdA show that lower amounts were removed than for TcdB, with Enterosgel removing approximately 40% after 60 min compared to 81% for Charcodote (p = 0.24). The detected concentration of the positive control samples for both toxins remained stable over the time course of incubation. The equilibrium adsorption isotherm in Fig. 1C displays q_e_, the amount of TcdB adsorbed (ng/g adsorbent), against C_e_, the residual concentration of TcdB in solution. Results indicate that Enterosgel has a capacity in excess of 34 µg/g for TcdB which is comparable with Charcodote (43 µg/g). For TcdA (Fig. 1D) Enterosgel had a capacity of 32 ng/g, whereas Charcodote displayed a larger capacity (112 ng/g). The data shows a large difference in the adsorption capacity for the two toxins, with a factor of 1000 times less removal of TcdA than TcdB.

**Figure 2:**
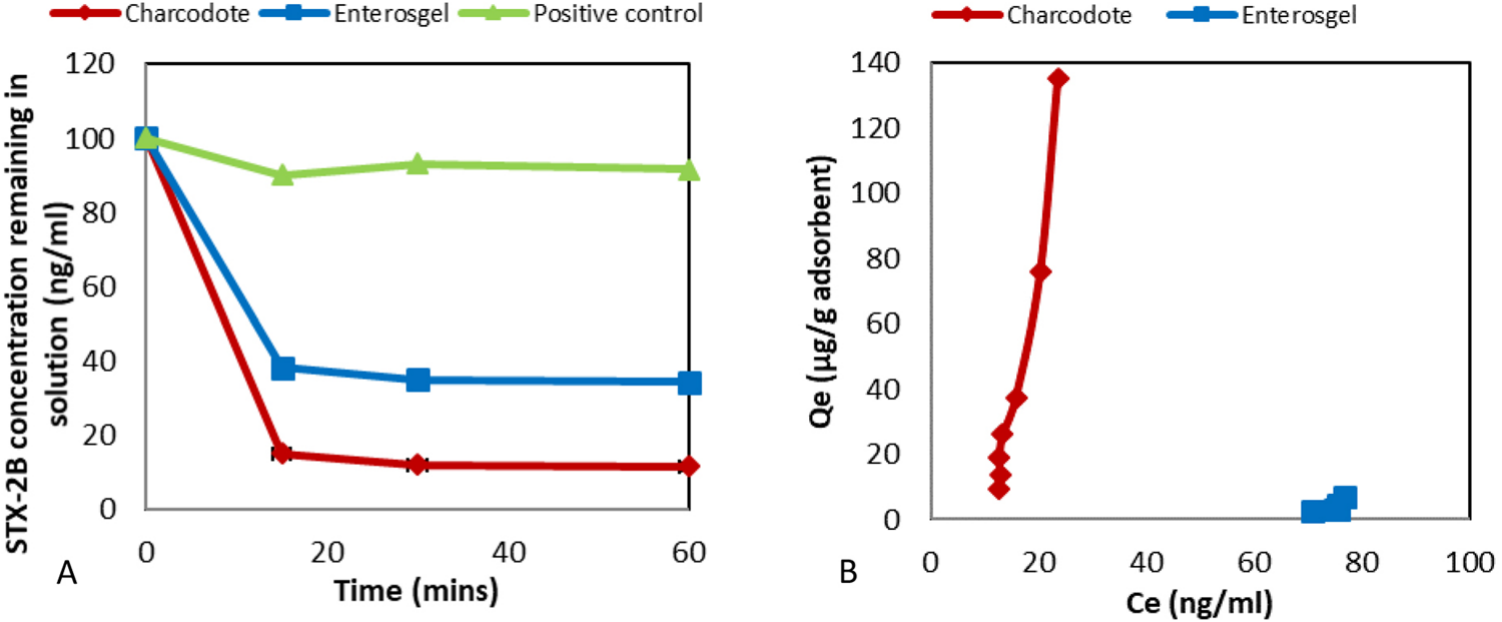
(**A**) Kinetic adsorption for Stx-2B showing remaining concentration over time for Enterosgel, Charcodote and positive control (no adsorbent) (mean ± sem). (**B**) Equilibrium adsorption isotherm (Qe) of Stx-2B against remaining concentration in solution (Ce) for Enterosgel and Charcodote.

Adsorption kinetics results (Fig. 2A) indicated that Enterosgel was able to adsorb approximately 60% of Stx-2B within the first 15 min of incubation, although levels of removal were less than for Charcodote (p = 0.54). Positive control sample concentrations also remained stable over the same time course. The equilibrium adsorption capacity (q_e_) of Enterosgel and Charcodote (Fig. 2B) was 6.7 µg/g and 135 µg/g respectively.

**Figure 3:**
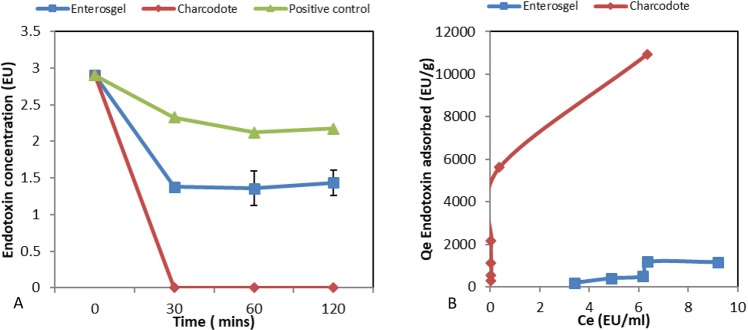
(**A**) Adsorption kinetics for endotoxin, showing remaining concentration (EU) over time for Enterosgel, Charcodote and positive control (no sorbent) (mean ± sem). (**B**) Equilibrium adsorption isotherm (Qe) of Endotoxin against remaining concentration in solution (Ce) for Enterosgel and Charcodote.

Adsorption kinetics results (Fig. 3A) indicated that Enterosgel was able to adsorb Endotoxin within the first 30 min of incubation although levels of removal were less than Charcodote which removed all endotoxin (p = 0.25). The Polymyxin B (positive control) reduced endotoxin levels close to zero, and the negative control measured no endotoxin. There was a reduction in the measured endotoxin concentration in the positive control sample (without adsorbent), which is not uncommon in endotoxin assays. The equilibrium adsorption capacity graph displayed in Fig. 3B shows that Enterosgel removed in excess of 1100 Endotoxin units (EU) (~165 ng) of Endotoxin, compared to 10900 EU (~1500 ng) for Charcodote. Table 1, shows the amount of each bacterial toxin that could be removed by a recommended single dose of Enterosgel (22.5 g) compared to a single dose of Charcodote (25–50 g).

**Table 1:** Quantity of each bacterial toxin and bile acid that can be removed by the recommended single dose of Enterosgel and Charcodote (Enterosgel 3 doses daily, Charcodote every 4–6 hours).

Sorbent	Enterosgel (single dose 22.5 g)	Charcodote (single dose 25–50 g)
*C*. *difficile* TcdA (ng)	739.1	2819–5638
*C*. *difficile* TcdB (µg)	764.0	1073–2146
Shiga toxin Stx2-B (µg)	151.0	3375–6750
Endotoxin (mg)	3.7	39.1–78.2
Taurocholic acid (mg)	13.5 (1.3%)	172.3 (17.2%)
Glycocholic acid (mg)	38.2 (3.8%)	349.6 (34.9%)
Taurochenodeoxycholic acid (mg)	32.3(3.2%)	390.9 (39.0%)
Glycochenodeoxycholic acid (mg)	104.9 (10.5%)	404.6 (40.5%)

In brackets the daily percentage removal of each bile acid (calculated using 2%w/v bile salt concentration, average 600 mL bile/day, approximate equal concentrations of each bile acid).

### Analysis of the adsorption kinetics for bacterial toxins

The rate of adsorption of the toxins by the adsorbents was analysed using the 1st-order rate kinetic model and the pseudo-2nd-order model, and the rate constants (k_1_ and k_2_) for each were calculated in addition to the correlation coefficient (R^2^) (Table 2 and Supplementary Figs S1–S4). The linearity of the plot and values of correlation coefficients indicate a better fit of pseudo-2nd-order model to the experimental data (higher R^2^ values), with exception of TcdA adsorption kinetics on both adsorbents, for which both 1^st^- and 2^nd^-order equations gave R^2^ > 0.99 (Supplementary Fig S1a,b).

**Table 2:** Lagergren 1st order rate constant K_1_ (min-1) and Pseudo-2nd-order rate constants K_2_ (g x mg-1 x min-1) for adsorption of C. difficile TcdA, *C*. *difficile* TcdB, Shiga toxin Stx2-B and Endotoxin by Enterosgel and Charcodote.

	Enterosgel				Charcodote			
	1^st^ Order rate		2^nd^ order rate		1^st^ Order rate		2^nd^ order rate	
	k_1_	R^2^	k_2_	R^2^	k_1_	R^2^	k_2_	R^2^
*C*. *difficile* TcdA	5.25E-03	0.9908	4.00E-04	0.9985	3.06E-03	0.9991	3.10E-04	0.9998
*C*. *difficile* TcdB	1.18E-02	0.8777	8.33E-04	1.000	9.51E-02	0.7905	1.66E-04	0.9957
Shiga toxin Stx2-B	9.20E-04	0.7277	1.85E-06	0.7805	9.20E-04	0.6928	3.23E-06	0.8898
Endotoxin	2.30E-04	0.5543	1.64E-02	0.9992	—	—	2.46	1.000

The assumptions used for derivation of these models differ only by the reaction rate order for the interaction between the adsorbate molecule and the localised adsorbent site. It should be noted that other assumptions, such as absence of interaction between the adsorbed molecules, independence of the energy of adsorption on surface coverage and the constant concentration of adsorbent sites, are applicable to the systems studied. The initial toxin concentrations were very low, and the adsorbed amounts were far below the monolayer surface coverage, justifying these assumptions. In the case of Stx-2B adsorption neither model produced satisfactory correlation with the experimental data for both adsorbents, suggesting a more complex mechanism of adsorption kinetics (Supplementary Fig S3a,b).

### Fitting adsorption isotherms equations to the experimental plots of adsorption of toxins

According to Giles classification of adsorption isotherms for adsorption from liquid phase, there are four main shapes commonly observed^22^, specifically focused on adsorption from liquid phase and this was used in our data interpretation: C-type, which is effectively Henry’s type isotherm described by a straight line; L-isotherm is described by Langmuir or Freundlich type equation; H-isotherm is a special case of L-isotherm with high affinity between adsorbate and adsorbent; and S-isotherm has sigmoid shape which reflects the existence of at least two mechanisms of adsorption (it has an inflection point). Linear regression analysis of all the experimental isotherms was conducted using a linear adsorption isotherm and linear forms of Langmuir and Freundlich two-parameter equations. The non-linear regression analysis was conducted using two-parameter Langmuir and Freundlich adsorption isotherm equations and three-parameter equations; Toth^23^, Langmuir -sigmoid^24^ and combined Langmuir-Freundlich^25^ equations (equations listed in Supplementary Data section S3).

The equilibrium adsorption isotherm plots for individual toxins for the both adsorbents (Supplementary Figs S5–10) have complex shapes. If the point (0;0) is taken into account, most of them have sigmoid shape (both Charcodote and Enterosgel with TcdB and Stx-2B and Enterosgel with endotoxin) and thus belong to S-type isotherms, Charcodote – endotoxin is best described as H-type isotherm and adsorption isotherms for both Charcodote and Enterosgel with TcdA have a more complex shape.

The linear regression analysis of the experimental data for each toxin and adsorbent showed poor correlation with linear forms of Langmuir or Freundlich equations, the highest R^2^ = 0.898 being for Enterosgel – endotoxin, whereas other correlation coefficients were below 0.8 (not included in this paper). Whereas, non-linear regression analysis using the two- and three-parameter adsorption isotherm equations gave various degrees of curve fit. The results of the curve fitting of the experimental data for both adsorbents are summarised in Table 3 (and Supplementary Data S3). For at least two sets of experimental data; adsorption of endotoxin by Enterosgel, and adsorption of Stx-2B by Enterosgel, none of the equations produced a good curve fit according to non-linear regression analysis. The Langmuir sigmoid equation gave R^2^ > 0.9 for most experimental data sets, however, it gave negative values for at least one parameter in curve fitting, which contradicts the physical meaning of these parameters. The Freundlich equation produced a good fit (R^2^ > 0.9) for four sets of data (Enterosgel- TcdA, TcdB and Charcodote – Stx-2B, TcdB). As expected, Charcodote - endotoxin adsorption isotherm of H-type was well described well by both Langmuir and Toth equations with high value of the adsorption constant K_L_ or K_T_, respectively. It should be noted that the experimental errors in these measurements were high due to the low concentrations of the solutes used.

**Table 3:** Parameters of curve fitting of the experimental data for Enterosgel (E) and Charcodote (C) for each of the bacterial toxins using the Henry (H), Langmuir (L) Freundlich (F), Toth (T), Langmuir -sigmoid (L-S) and combined Langmuir-Freundlich (L-F) equations listed in Supplementary Data section S3.

Adsobent-Adsorbate	C- end-otoxin	E- end-otoxin	C- TcdA#	E- Tcd A	C-Stx-2B†	E- Stx-2B†	C- TcdB	E- TcdB
**Equation**								
H	‡	‡	‡	‡	‡	‡	‡	‡
K_H_	1.41.10^3^	86.8	—	2.117	3.303	0.055	1.049	0.658
R^2^	0.504	0.686	—	0.740	0.558	0.600	0.881	0.745
L	**√**	‡	‡ §	§	§	§	§	§
a = q_m_K_L_	**4**.**27**.**10**^**5**^	83.4	neg	7.312	0.928	0.0022	0.549	0.213
b = K_L_	**4**.**79**	neg	neg	neg	neg	neg	neg	neg
R^2^	**0**.**968**	0.687	0.859	0.952	0.980	0.899	0.932	0.927
F	‡	‡	‡ §	**√**	**√**	‡	**√**	**√**
K_F_	5.04.10^3^	68.0	71.3	**6**.**038**	**0**.**0028**	6.41.10^−8^	**0**.**0608**	**4**.**40**.**10**^**−4**^
1/n	0.315	1.126	—	**0**.**481**	**3**.**41**	4.165	**1**.**839**	**3**.**12**
R^2^	0.886	0.690	0.797	**0**.**952**	**0**.**9906**	0.700	**0**.**932**	**0**.**940**
T	**√**	‡	§	‡	§	§	‡	§
a	**4**.**48**.**10**^**5**^	3950	—	29.3	141	130	1.117	42.3
b	**4**.**64**	2010	—	0.974	neg	neg	2.21.10^−5^	−39.7
t	**0**.**955**	2.00	—	0.500	6.568	1.778	0.0004	2.32
R^2^	**0**.**968**	0.686	—	0.834	1.000	0.967	0.8814	1.000
L-S	§		§	§	§	§	§	§
a	0.751	neg	neg	neg	neg	1.16.10^5^	neg	4.784
b	neg	0.0087	0.027	0.146	0.0129	neg	0.139	neg
c	neg	0.441	0.178	0.439	19.1	neg	136	neg
R^2^	0.904	0.807	0.908	0.952	0.990	0.967	0.939	0.948
L-F	**√**	§	‡	§	§	‡ §	§	§
a	**4**.**04**.**10**^**5**^	0.106	—	—	103	—	2.412	2.538
b	**4**.**50**	0.0001	—	—	−0.0026	—	−2.156	−1.917
n	**0**.**977**	5.571	—	—	2.93	—	0.028	0.0734
R^2^	**0**.**968**	0.774	—	—	0.992	—	0.963	0.936

neg – negative value of the parameter; - no meaningful approximation.

^†^These data were also fit with the Sigmoidal Hill three-parameter equation: y = ax^n^/(c^n^ + x^n^): a = 1.23.10^6^, c = 174 and n = 3.41, R^2^ = 0.989.

^√^The equation fits the experimental data with R^2^ > 0.9.

^‡^The equation does not fit the experimental data.

^§^The equation fits the experimental data but the negative value for b means also negative values for q_m_ or K_L_, or both, which does not have a physical meaning.

^#^These data fit with the three-parameter equation: y = x/(a + bx^n^), a = 0.024, b = 0.00826, n = 2.36, R^2^ = 0.925.

### Adsorption of bile acids by Enterosgel

**Figure 4:**
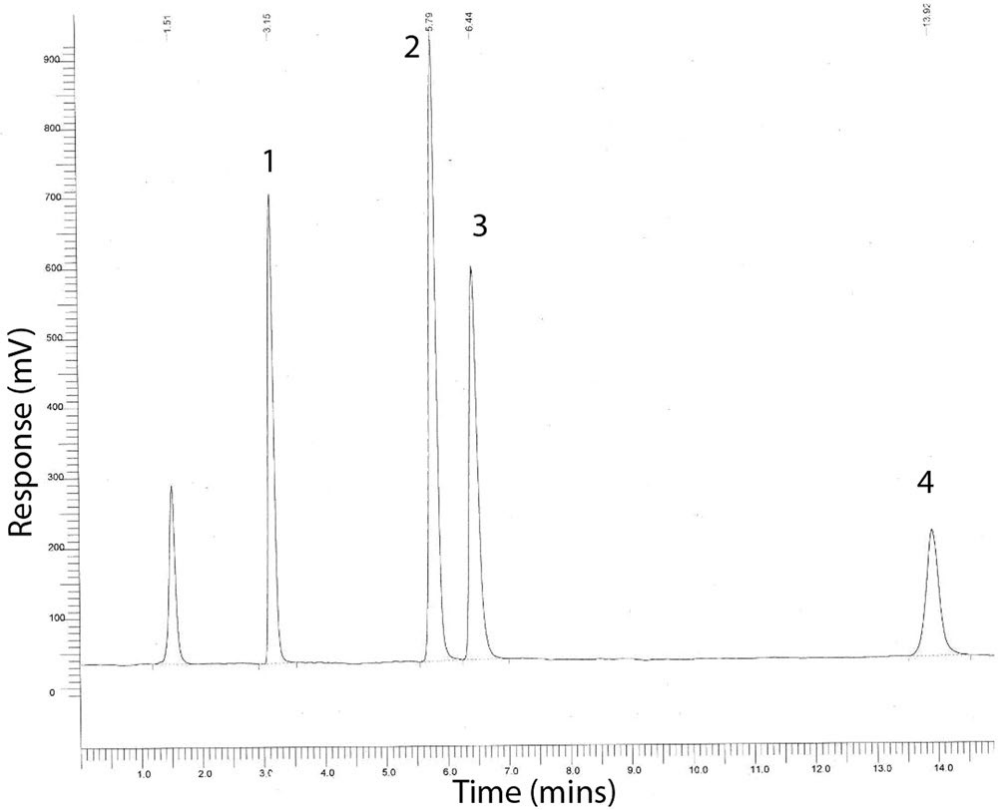
Representative chromatographic trace with a solution of 125 µg/mL Taurocholic acid (1, 3.15 mins), and 250 µg/mL Glycocholic acid (2, 5.79 mins), Taurodeoxycholic acid (3, 6.44 mins) and Glycochenodeoxycholic acid (4, 13.92 mins).

The representative HPLC chromatographic trace in Fig. 4, shows the retention times of a mixed solution of 125 µg/mL 1. Taurocholic acid (3.15 min), and 250 µg/mL 2. Glycocholic acid (5.79 min), 3. Taurochenodeoxycholic acid (6.44 min) and 4. Glycochenodeoxycholic acid (13.92 min). The individual calibration curves of Log concentration against Log peak area gave R^2^ values respectively of 0.998, 0.9937, 0.9986 and 0.9984. Adsorption kinetic results over 120 min demonstrate that Enterosgel removed 8% of Taurocholic and Taurochenodeoxycholic, 13% Glycocholic and 27% of Glycochenodeoxycholic compared to 100% removal by Charcodote, from a solution containing 250 µg/mL Taurocholic acid and 500 µg/mL of the remaining bile acids. Table 1 shows the amount of each Bile acid that could be removed by the recommended single dose (22.5 g) of Enterosgel, and the theoretical daily percentage removal of each bile acid from the body.

### Adsorption of Cetirizine hydrochloride and Amitriptyline hydrochloride by Enterosgel

**Figure 5:**
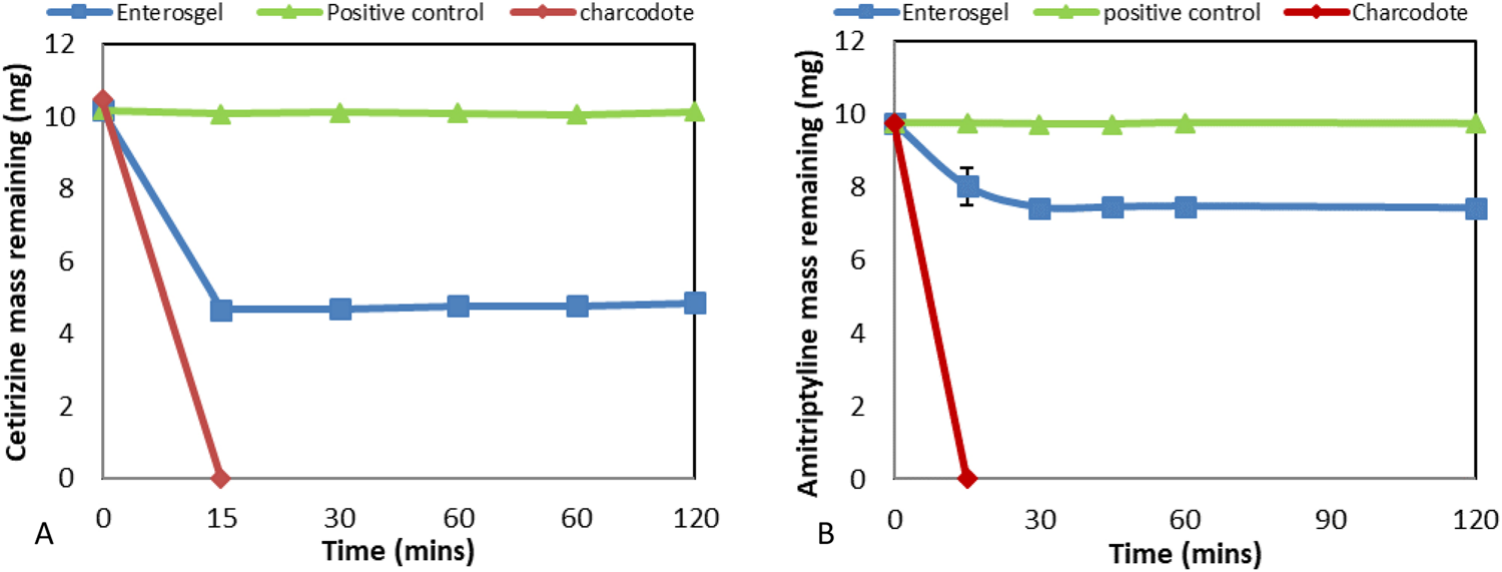
Kinetic adsorption for Cetirizine hydrochloride (**A**) and Amitriptyline hydrochloride (**B**), showing remaining mass (mg) over time for Enterosgel, Charcodote and positive control (no adsorbent) (mean + sd).

Calibration curves for Cetirizine hydrochloride (in the range of 0–65 µg/mL) and Amitriptyline hydrochloride (0–30 µg/mL) were analysed for a linear fit and gave an R^2^ value of 0.999, 0.993 respectively. Kinetic adsorption results (Fig. 5A) indicated that Enterosgel adsorbed 53% of Cetirizine hydrochloride within the first 15 min of incubation and this level remained stable over the 120 min incubation, in contrast to Charcodote which removed 100% within the first 15 min. The positive control sample with no sorbent present remained stable. Adsorption kinetics results (Fig. 5B) for Amitriptyline hydrochloride indicated that Enterosgel adsorbed 23% at 30 min which remained stable over the time course whereas Charcodote also removed 100% within the first 15 min.

## Discussion

The adsorption efficacy and capacity of Enterosgel was compared to Charcodote for bacterial enterotoxins and endotoxin associated with gastrointestinal infection and known causes of complications such as diarrhoea. Activated carbons have been used extensively to treat poisoning and other conditions such as diarrhoea, with evidence supporting their mechanism as adsorption of target substances from the gut^26^. Similarly, other oral adsorbents that are silica based such as Diosmectite (Cf Pharma Ltd, Ireland) suggest diarrhoeal treatment is achieved through the physicochemical properties of silica materials which have adsorptive activity against a range of substances in the gut^27^. Enterosgel is an organic silicon based material and therefore like most oral adsorbents its mechanism of action is by physical adsorption. However, it also has both surface hydrophilic groups (-OH) and hydrophobic groups (-CH_3_) enabling adsorption of both hydrophilic and hydrophobic substances^16^.

Overall results in Table 1, demonstrate Enterosgel has substantial adsorption capacity for TcdA, TcdB, Stx2-B and endotoxin *in vitro*, although capacity it is lower than for Charcodote. Enterosgel like activated carbon contains both micropores (<2 nm) and mesopores (2–50 nm), enabling adsorption and retention of small molecules in the micropores and larger toxins in the mesopores. The reported pore size distribution depends on the water content but the average pore diameter is 20 nm, which makes this material suitable for adsorption of large bacterial toxins. The difference in the capacity data between the two materials is not unexpected as Enterosgel has a specific surface area of 200 m^2^/g (dry weight) available for adsorption^16^, which is a factor of ~10 times less than activated carbon.

Enterosgel adsorbed the two main virulence factors of *Clostridium difficile;* TcdA and TcdB, which can cause inflammation and damage to the mucosa in the colon resulting in diarrhoea^28^. Adsorption capacity for TcdA was a factor of 1000 times less than TcdB, which may be explained in part by the larger size of the TcdA molecule, 307 kDa compared to 64 kDa for TcdB, and the nature of the two molecules. TcdA was a native full length protein derived from the organism, whereas TcdB was a recombinant *E*. *coli* derived enzymatic portion of the tripartite structure. Enterosgel adsorbed Stx2-B, produced by the bacteria *S*. *dysenteriae* and the Shigatoxigenic group of *E*. *coli*. Stx2-B is a 10 kDa pentamer that binds to specific glycolipids on the host cell and can cause symptoms such as gastroenteritis. Similarly, Enterosgel adsorbed endotoxin derived from *E*. *coli* stain 055:B5 which is 10 kDa in size, although it readily forms larger aggregates up to 1000 kDa, and can cause impaired intestinal absorption, resulting in diarrhoea^29^. The positive control samples for TcdA, TcdB and Stx-2 with no adsorbent present demonstrated that the toxins were not degrading over the time course or binding non-specifically. However, the was a degree of reduction in the endotoxin positive control over time, the LAL assay suffers from inhibition interference and a number of contributing factors such as pH and divalent cations could account for this drop.

The adsorption kinetic analysis in Table 2, showed a better fit of pseudo-2nd-order model to the experimental data, suggesting the adsorption of toxins by Enterosgel and Charcodote follows a second order reaction pathway.

These findings are supported by Fluer *et al*.^30^, who showed that Enterosgel has the ability to remove staphylococcal enterotoxins A and B from biological substrates and reduce bacterial growth. They suggest that this ability to remove (adsorb) bacterial toxins is one of the possible mechanisms of its therapeutic action. Other possible mechanisms of the therapeutic action of enterosorption include immunocorrection^31^, creation of an unfavourable environment for pathogenic microorganisms^32^, and inhibiting viral replication^33^ however, in all cases the primary action is physical adsorption. For Enterosgel, the adsorption of molecules occurs via the diffusion of molecules within the three-dimensional polymer network filled with water and their retention by the polymer chains.

Results in Table 1 also demonstrate that Enterosgel can adsorb small molecules such as bile acids *in vitro*. In line with other studies evaluating the capacity of Enterosgel for small molecular weight substances, adsorption was significantly lower than activated carbon due in part to its greater surface area and micropore volume, whereas, Enterosgel shows increasing adsorption capacity with the increase in the molecular weight of the solute^16,19^.

Excess bile acid concentration in the colon is often a cause of chronic diarrhoea and 30% of IBS-D patients have associated Bile Acid Malabsorption, which are currently treated by bile acid sequestrants^34^. There are various possible mechanisms by which enterosorbents could improve IBS-D symptoms, including adsorption of mediators such as histamine and serotonin that are postulated to play a causative role in IBS, and adsorption of bacterial products and bile acids, which also have been implicated in the generation of IBS symptoms^35,36^. The immune proteins released, the bacterial breakdown products of the micro-environment, together with fat molecules and bile acids can all contribute to the development of diarrhoea in these patients. Although Enterosgel showed only moderate to low adsorption of these bile acids (1.3–10.5%), in combination with removal of other potential targets, it could be of therapeutic benefit to patients with IBS-D. A study by Tkachenko *et al*., 2015 investigating Enterosgel use in IBS-D patients, showed both a significant improvement in reduction in stool frequency per week and improvement in normalisation of stool (improvement in Bristol stool scale)^9^. Similarly, in a more recent study the in treatment of IBS-D, there was observed a clinical improvement in the health of patients and a decrease in the frequency of bowel movements^12^.

One disadvantage of enterosorption is the potential for interaction with orally administered medications and disruption of their pharmacokinetics, during their co-residence in the gastrointestinal tract. In this study the two medications Cetirizine hydrochloride and Amitriptyline hydrochloride were selected as these are commonly administered to patients with the indicated condition IBS and allergies (indicated use outside EU). Cetirizine and Amitriptyline are both delivered as 10 mg tablets, the results show that a single dose of Enterosgel (22.5 g) could potentially adsorb 5.7 mg and 2.3 mg respectively, if administered concomitantly. However, Cetirizine is adsorbed rapidly and effectively, with peak serum levels reached only one hour after administration of a 10 mg orally administered dose^37^. Likewise, Amitriptyline is readily adsorbed from the gut and it is likely that other interfering factors such as the presence of food would limit this optimal adsorption^38^. Other studies investigating the interaction of Enterosgel with drugs have shown the adsorption capacity towards these low molecular mass substances by at least an order of magnitude lower than activated carbon^19–21^. The group of Koya *et al*.^39^, developed a model for estimating the risk of drug interaction in clinical practice for oral carbon adsorbent AST-120. They concluded, that the detrimental effects of concomitantly administered drug adsorption by enterosorbent on both drug efficacy and pharmacokinetics could be diminished by increasing the interval between administrations. The advantageous lower level of adsorption of Cetirizine and Amitriptyline and the recommended 2 hour window between patient dose of either medicine and Enterosgel administration will be beneficial in limiting Enterosgels influence on their bioavailability and drugs with similar profiles.

In conclusion, the *in vitro* adsorption results investigated in this study suggest that the therapeutic mode of action of Enterosgel as a treatment for diarrhoea, is via the adsorption of target substances such as bacterial toxins and excessive bile acids from the gut, followed by complete removal from the body. This study also highlights the limitation of enterosorbents due to their potential for interaction with medications, although results indicate that Enterosgel adsorbs significantly less than activated carbon and support the rational for their use at least 2 hours after taking medications to prevent influencing bioavailability.

These promising results advocate expanding the use of oral adsorbents in medicine, particularly in prevention and control of the spread of antibiotic resistance. There is need for more research into intestinal adsorbents, their mechanism of action and their potential use as an additive treatment or alternative to antibiotics, via their removal of pathogenic bacterial toxins and other substances in the GIT. Another important area is prevention and treatment of chronic non-infectious diseases, the development of which play an important role in intestinal flora and endotoxemia.

## Methods

### *Clostridium difficile* toxin A and B adsorption by Enterosgel

Triplicate samples of Enterosgel (Bioline Products s.r.o, Czech Republic) and commercial control - activated carbon Charcodote (Teva UK Ltd) (5%w/v) were incubated at 37 °C in a shaking incubator set at 120 rpm with recombinant *Clostridium difficile* Toxin B (TcdB) (BioTechne, UK) at a concentration of 100 ng/mL in phosphate buffered saline with 1% bovine serum albumin (PBSB), or natural *Clostridium difficile* Toxin A (TcdA) (Abcam, UK) at a concentration of 10 ng/mL in PBSB. At selected time points (120 min) samples were centrifuged at 9000 rpm for 2 min and aliquots collected and measured for TcdB or TcdA concentration using a Premier Toxins A + B kit (Launch Diagnostics, UK). Controls consisted of a positive control for each toxin in buffer with no sorbent present and negative buffer control. The equilibrium adsorption capacity of Enterosgel for TcdB and nTcdA was then determined using a range of material weights (3.7–26 mg) incubated with 100 ng/mL TcdB or 10 ng/mL TcdA in PBSB, for 3 h at 37 °C.

### Shiga Toxin II adsorption by Enterosgel

Enterosgel and Charcodote (1.33%w/v, n = 3) were incubated as above with Enterohemorrhagic *E*. *coli* (EHEC) Shiga Toxin II subunit B (Stx-2B) (Sino Biological Inc, UK) at a concentration of 100 ng/mL in PBSB. Stx-2B concentration was measured using an EHEC Stx-2B Enzyme linked immunoadsorbent assay (Sino Biological Inc, UK). Controls consisted of a positive control of EHEC Stx-2B in PBSB with no sorbent present and negative buffer control. The equilibrium adsorption capacity of Enterosgel for Stx-2B was then determined using a range of material weights (1.3–35 mg) incubated with 100 ng/mL of Stx-2B for 3 h at 37 °C.

### *Escherichia coli* endotoxin adsorption by Enterosgel

Enterosgel and heat sterilised Charcodote (5% and 2.5%w/v respectively, n = 3) were incubated at 37 °C in a shaking incubator set at 120 rpm with Control standard Endotoxin from *E*. *coli* (Charles River Laboratories, UK) at a concentration of 5EU/ml in simulated intestinal fluid (SIF) prepared according to the British Pharmacopoeia^40^. At selected time points samples were vortexed for 60 s then centrifuged at 9000 rpm for 2 min and aliquots collected. Standards (Control standard Endotoxin 0–50 EU in water) and samples were measured for endotoxin concentration using a Limulus amebocyte lysate endosafe endochrome-K colorimetric kinetic assay (Charles River Laboratories, UK) on a Biotek kinetic plate reader. Controls consisted of a positive control of 5EU Endotoxin in SIF, Polymyxin B (2.5%w/v) incubated with 5EU endotoxin and a negative control of SIF. The equilibrium adsorption capacity of enterosorbents was then determined using a range of material weights (1.2–52 mg) incubated with 25EU of Endotoxin in SIF for 3 h at 37 °C.

### Adsorption kinetics and isotherms

Equilibrium adsorption isotherm plots were calculated for the adsorption quantity (${q}_{e}$) calculated using the equation below, against remaining concentration in solution (C_e_) after incubation with each adsorbent for 3 hours.

$${q}_{e}=(({{\rm{C}}}_{0}-{{\rm{C}}}_{{\rm{e}}})\ast {\rm{V}})/{\rm{m}}.$$

where C_0_- initial concentration of solution; mg/L, C_e_- concentration after sorption; mg/L and V - volume; L and m-mass of the sorbent; g. Corrected plots were also generated taking into account the experimental drop in C_0_- concentration of solution over time.

Adsorption kinetics is often described by pseudo-first or pseudo-second order kinetics, which use the same assumptions with the only difference that adsorption is governed by first- or second-order reaction respectively. The rate of adsorption of each toxin by the adsorbents was analysed using the 1st-order rate kinetic model outlined by Lagergren^41^ expressed as:

$$\mathrm{log}({q}_{e}-q)=\,\mathrm{log}({q}_{e})-\frac{{k}_{1}t}{2.303}$$

where q and *q*_*e*_ are the values of adsorption quantity (ng/g) at time t and equilibrium, respectively. The graph of log (*q*_*e*_ − *q*) vs. t (min) was plotted for each toxin with the linearity indicating whether the adsorption kinetics follows the 1st-order mechanism. The rate constants (k_1_) for each adsorbate were calculated from the linear least square method in addition to the correlation coefficient (R^2^). The pseudo-2nd-order model also provides information on the kinetics of the adsorption process. We applied the linearized pseudo-2nd-order equation expressed as^42^:

$$\frac{t}{q}=\frac{1}{{k}_{2}\,{q}_{e}^{2}}+\frac{1}{{q}_{e}}t$$

The graph of t/*q* versus t (min) was plotted for each toxin and the rate constants (k_2_) and correlation coefficient (R^2^) calculated.

### Bile acid adsorption by Enterosgel

The method of Sardella *et al*.^43^, for the detection of bile acids using an HPLC with evaporative light scattering detector (ELSD) was adapted for detection of the conjugated bile acids (Glycocholic and Taurocholic acids) and the secondary bile acids (Taurodeoxycholic and Glycochenodeoxycholic acids) (Sigma Aldrich, UK). All the reagents were of analytical grade. HPLC measurements were made on a WATERS 717 plus Autosampler, PERKIN ELMER Series 200 Column oven, PE NELSON 900 series interface and PERKIN ELMER Series 200 LC pump. A PHENOMENEX, Luna 3 µ C18, 150 × 4.6 mm column was used as the analytical column and a Varian 385-LC ELSD evaporative light scattering detector was utilized for the detection. The adopted ELSD conditions were: 30 °C nebulisation temperature, 50 °C evaporation temperature, 1.7 L/min gas flow rate (Nitrogen) and 5.0 as the gain factor. The mobile phase was 50 mM ammonium formate in water/acetonitrile 60/40 (v/v) solution adjusted to pH 3.5. The analysis was carried out at a 1.0 mL/min eluent flow rate, 30 °C column temperature, 30 µL injection volume, and 15 min run time. Triplicate samples of Enterosgel and Charcodote (3.3%w/v) were incubated at 37 °C in a shaking incubator set at 120 rpm with a solution of 250 µg/mL Taurocholic, and 500 µg/mL Glycocholic, Taurochenodeoxycholic and Glycochenodeoxycholic acid in water. At selected time points (up to 120 minutes) samples were centrifuged and filtered through a 2 µm PTFE filter and diluted before analysis. Controls consisted of a positive control of bile acids with no adsorbent present and a negative control of water only.

### Pharmaceutical drugs; Cetirizine hydrochloride and Amitriptyline hydrochloride adsorption by Enterosgel

Triplicate samples of Enterosgel or Charcodote (22.5 g) were incubated in a 37 °C shaking incubator set at 120 rpm with 250 mL of Cetirizine hydrochloride or Amitriptyline hydrochloride at a concentration of 40 µg/mL in water (equivalent to 10 mg tablet). At selected time points (15, 30, 60, 120 min) samples were centrifuged at 9000 rpm for 2 min and aliquots collected and diluted 1:1.6 in water and measured for drug concentration by UV-spectroscopy. Controls consisted of a positive control of drug (40 µg/mL) with no adsorbent present.

### Statistics

Data are the mean ± sem of a single experiment with triplicate samples, with p values determined by parametric unpaired Student’s t test and considered significant when p ≤ 0.05. Calibration curves were analysed by Graph Pad Prism. The non-linear regression analysis was conducted using Curve Expert Professional 2.6.5* and SigmaPlot 14 software.

## Supplementary information

Supplementary Information

## Data Availability

The datasets generated during and/or analysed during the current study are available from the corresponding author on reasonable request.
