# Supplementary material for: Investigation of the adsorption capacity of the enterosorbent Enterosgel for a range of bacterial toxins, bile acids and pharmaceutical drugs
Source: Sci Rep. 2019 Apr 4;9:5629. doi: 10.1038/s41598-019-42176-z (PMC6449336; doi:10.1038/s41598-019-42176-z)
Supplement: Supplementary file 1 — Supplementary Information [file 41598_2019_42176_MOESM1_ESM.pdf]

Supplementary information.

Investigation of the adsorption capacity of the enterosorbent Enterosgel for a range of bacterial toxins, bile acids and pharmaceutical drugs.

Carol A. Howell, Sergey V. Mikhalovsky, Elena N. Markaryan, and Alexander V. Khovanov

S1. Adsorption kinetic analysis - 1st-order rate kinetic model and the pseudo-2nd-order model

The rate of adsorption of the toxins by the adsorbents was analysed using the 1st-order rate kinetic model proposed by Lagergren [S1] and the pseudo-2nd-order model and the rate constants (k_1 and k_2) for each were calculated in addition to the correlation coefficient (R^2).

Fig S1a. 1st-order rate kinetic model plot for TcdA

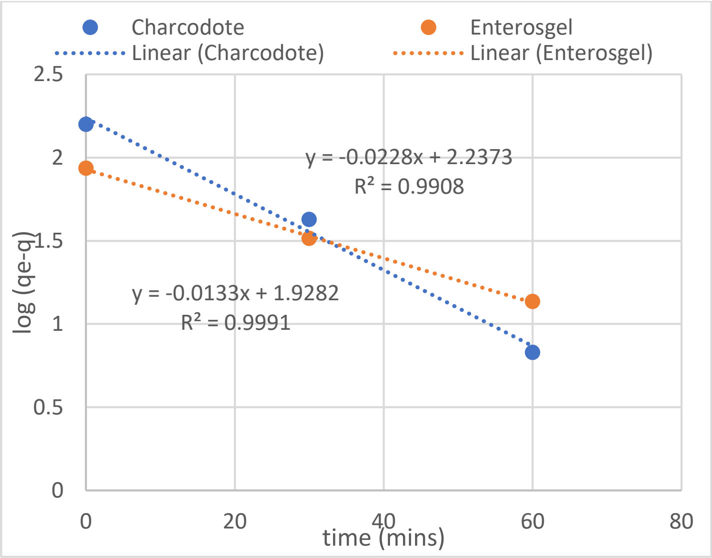

Fig S1b. pseudo-2nd-order rate kinetic model plot for TcdA

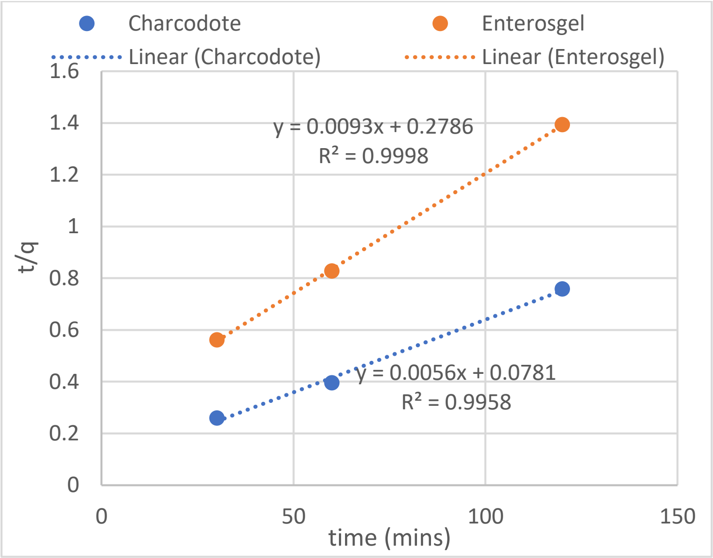

Fig S2a. 1st-order rate kinetic model plot for TcdB

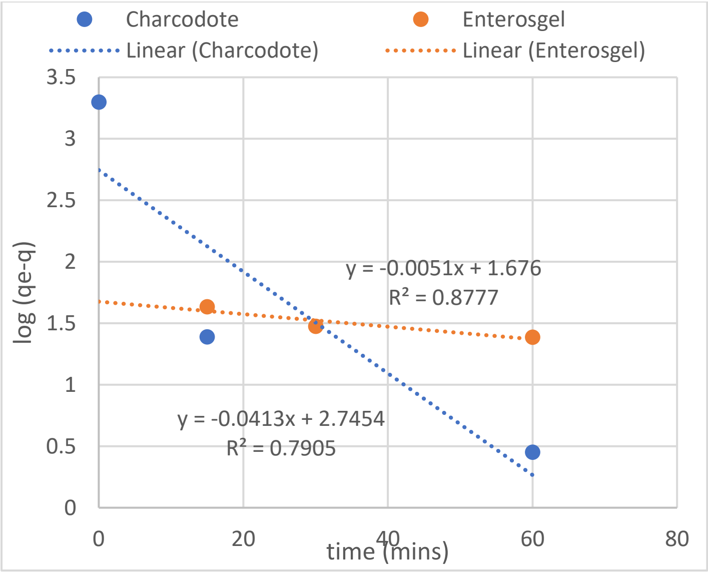

Fig S2b. pseudo-2nd-order rate kinetic model plot for TcdB

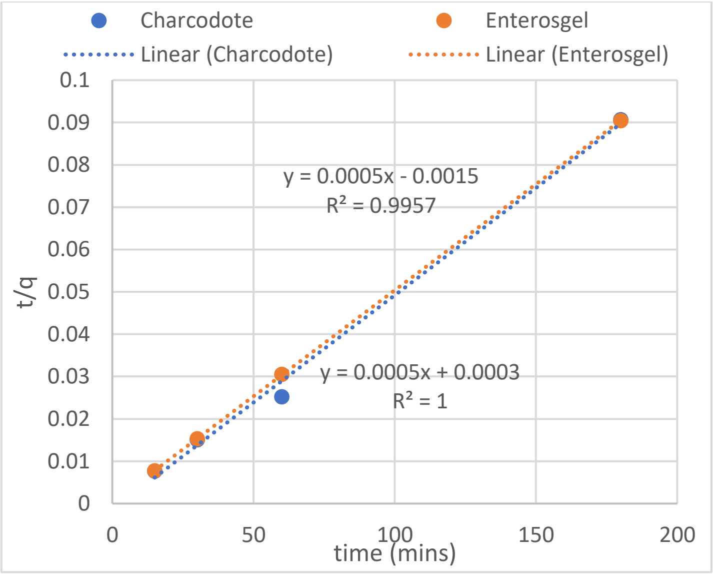

Fig S3a. 1st-order rate kinetic model plot for Stx-2B

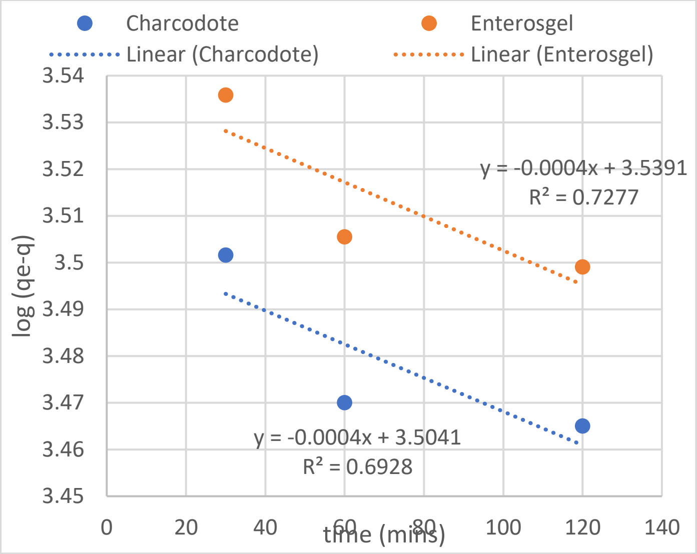

Fig S3b. pseudo-2nd-order rate kinetic model plot for Stx-2B

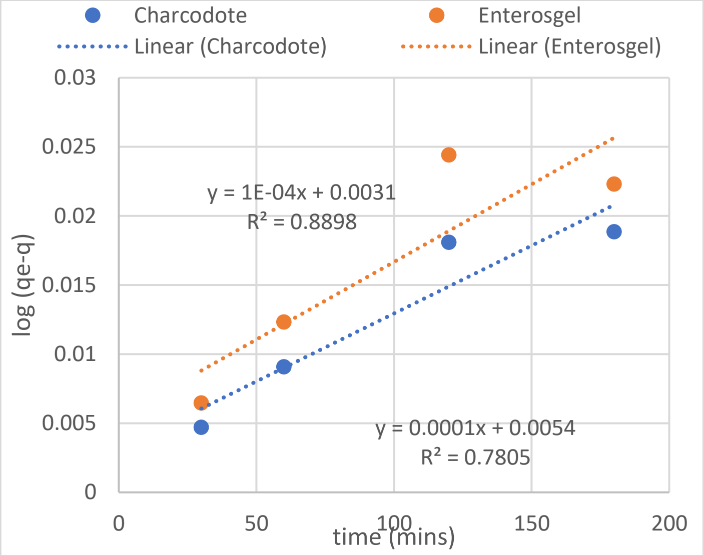

Fig S4a. 1st-order rate kinetic model plot for endotoxin

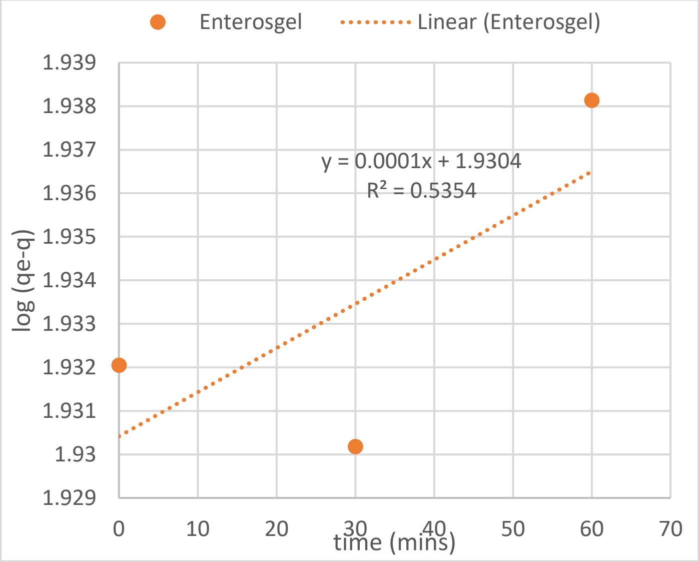

Fig S4b. pseudo-2nd-order rate kinetic model plot for endotoxin

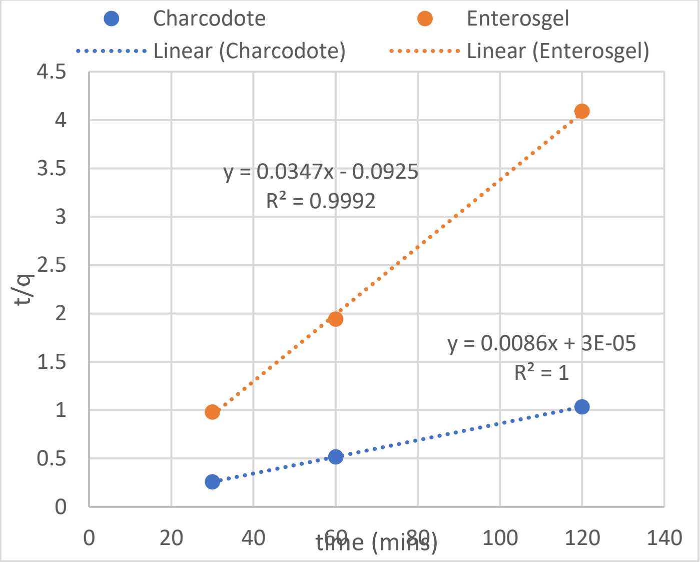

S2. Adsorption isotherm analysis

Fig S5a. Charcodote adsorption isotherm for TcdA

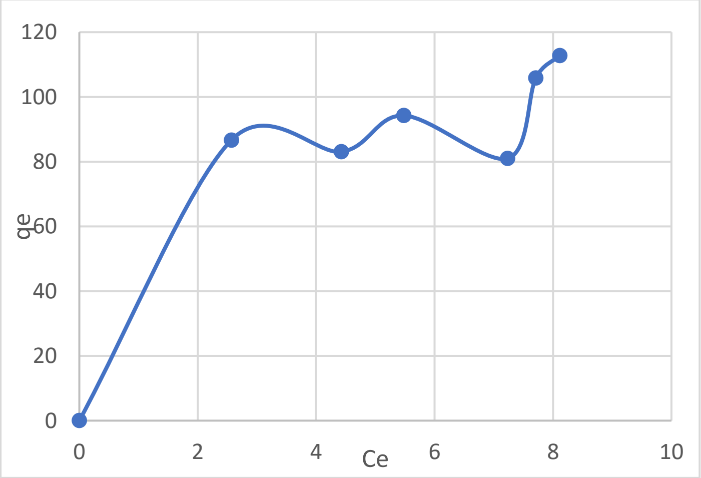

Fig S5b. Charcodote adsorption isotherm (corrected for C_0) for TcdA*

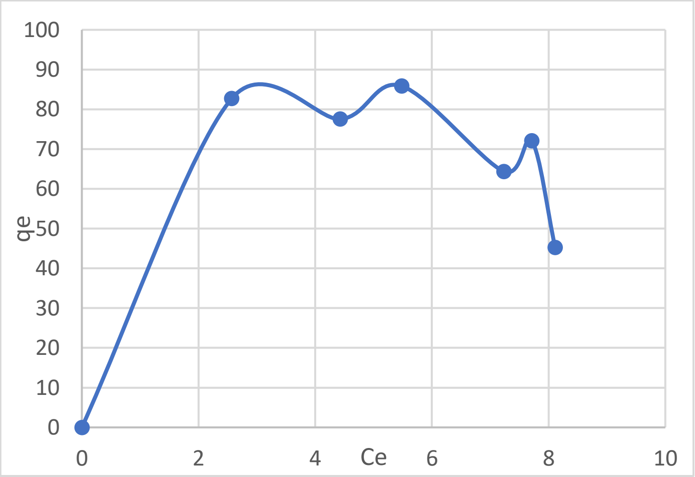

*In the control experiments without any adsorbent, a reduction of C_0 by approximately 3% (from 8.57 to 8.30 ng/mL) was observed. In Figures 5b and 6b the adsorbed amount q_e was corrected for the reduction of C_0 in the control.

Fig S6a.Enterosgel adsorption isotherm for TcdA**

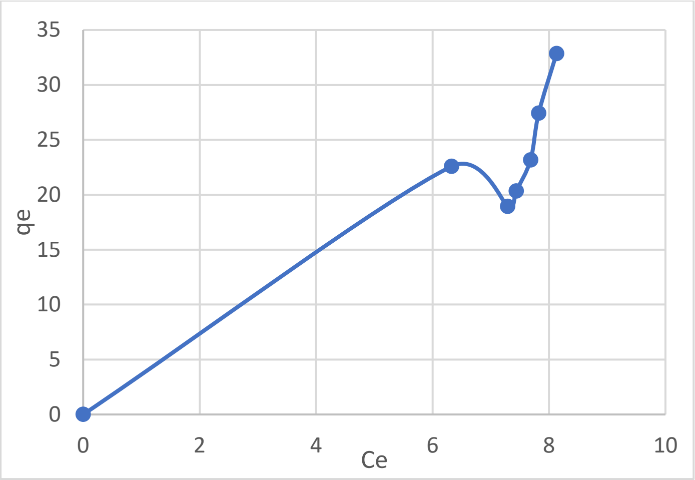

Fig S6b. Enterosgel adsorption isotherm (corrected for C_0) for TcdA*

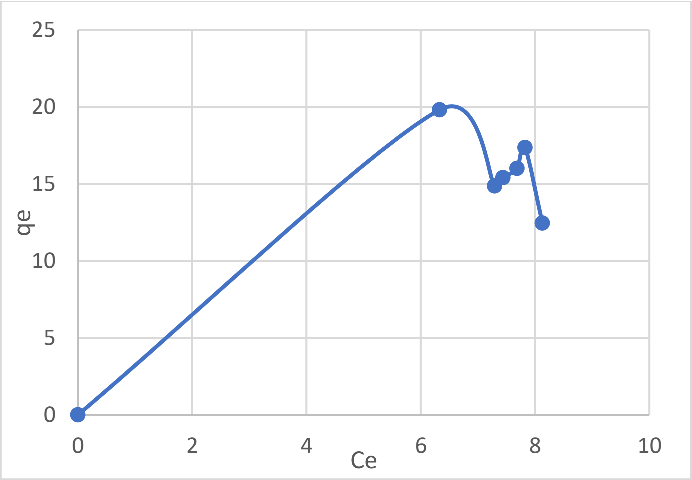

*In the control experiments without any adsorbent, a reduction of C_0 by approximately 3% (from 8.57 to 8.30 ng/mL) was observed. In Figures 5b and 6b the adsorbed amount q_e was corrected for the reduction of C_0 in the control.

Fig S7a. Charcodote adsorption isotherm for TcdB*

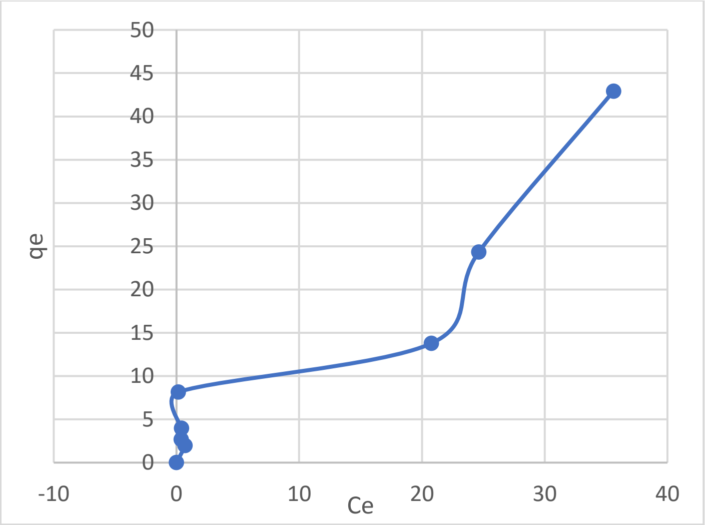

Fig S7b. Enterosgel adsorption isotherm for TcdB*

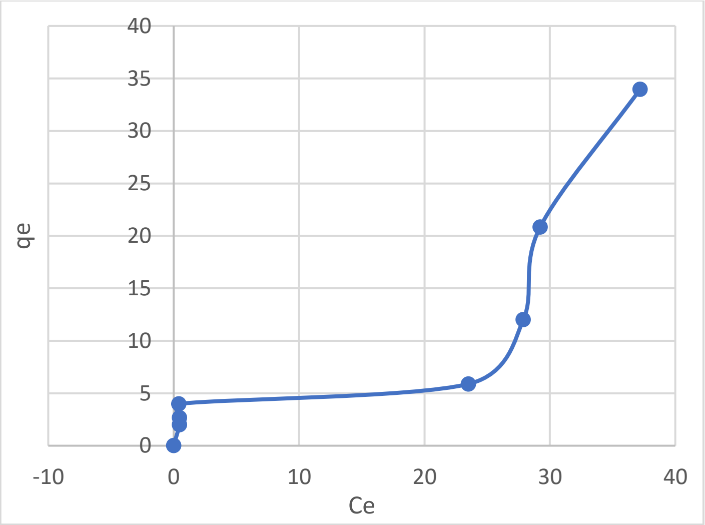

*C_0 remained constant over the course of the experiment.

Fig S8a. Charcodote adsorption isotherm Stx-2B*

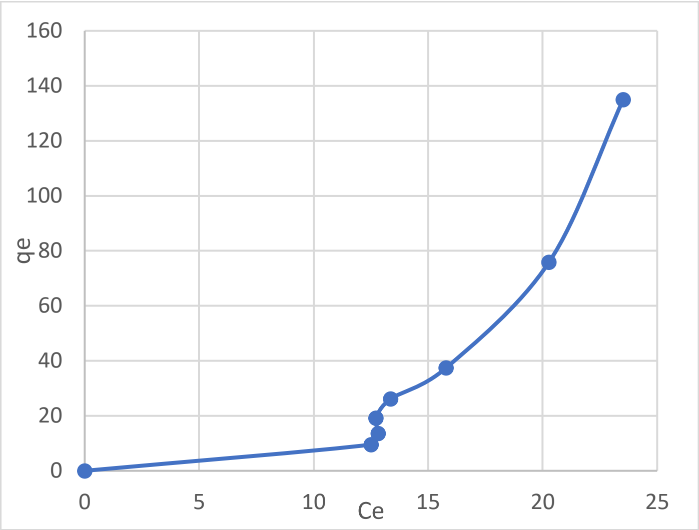

Fig S8b. Enterosgel adsorption isotherm Stx-2B*

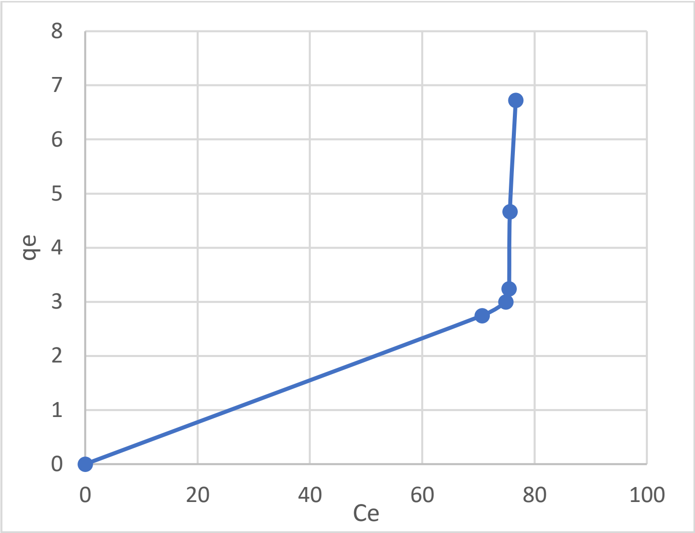

*C_0 remained constant over the course of the experiment.

Fig S9a.Charcodote adsorption isotherm for endotoxin

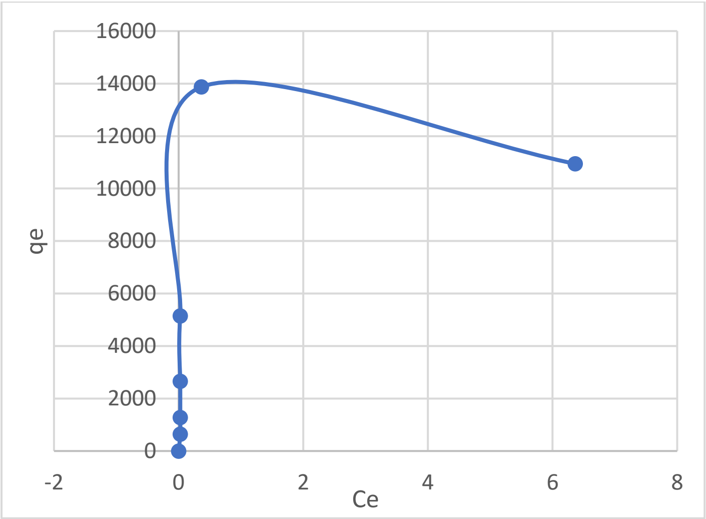

Fig S9b. Charcodote adsorption isotherm for endotoxin (corrected for C_0)*

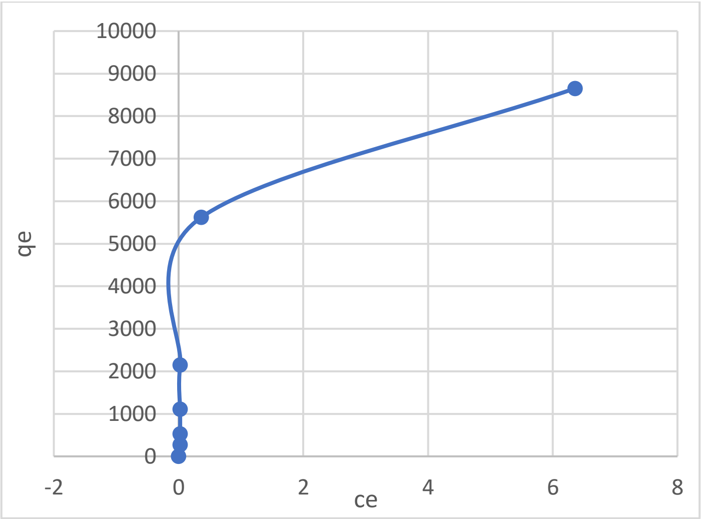

*In the control experiments without any adsorbent, a reduction of C_0 by approximately 11% (from 12.9 to 11.6 EU/mL) was observed. In Figures 9b and 10b the adsorbed amount q_e was corrected for the reduction of C_0 in the control.

Fig S10a.Enterosgel adsorption isotherm for endotoxin

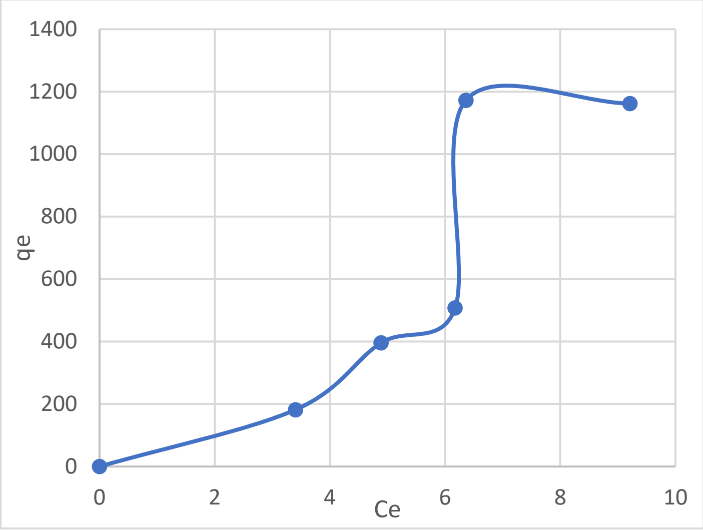

Fig S10b. Enterosgel adsorption isotherm for endotoxin (corrected for C_0)*

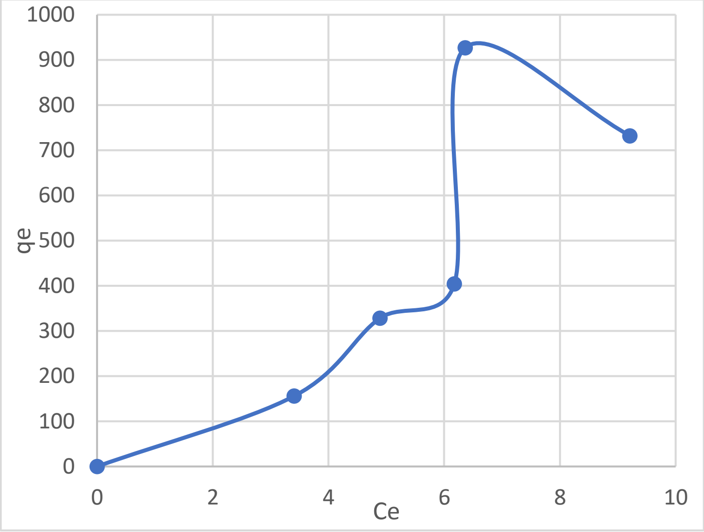

*In the control experiments without any adsorbent, a reduction of C_0 by approximately 11% (from 12.9 to 11.6 EU/mL) was observed. In Figures 9b and 10b the adsorbed amount q_e was corrected for the reduction of C_0 in the control.

S3. Fitting adsorption isotherms equations to the experimental plots of adsorption of toxins

According to Giles classification of adsorption isotherms for adsorption from liquid phase, there are four main shapes commonly observed [Giles, C.H., Smith, D., Huitson, A., 1974. A general treatment and classification of the solute adsorption isotherm. I. Theoretical. J. Colloid Interf. Sci. 47, 755–765]:

C-type, which is effectively Henry's type isotherm described by a straight line;

L-isotherm is described by Langmuir or Freundlich type equation; it has concave shape (or mathematically, convex upward or concave downward);

H-isotherm is a special case of L-isotherm with high affinity between adsorbate and adsorbent;

S-isotherm has sigmoid shape which reflects the existence of at least two mechanisms of adsorption. It has an inflection point.

The plots of adsorption isotherms obtained in this study have complex shape and suggest for most of them S-shape if the point (0;0) is taken into account (both Charcodote and Enterosgel with C.diff B and Shigella and for Enterosgel – E.coli), H-shape for Charcodote – E.coli and a more complex shape for both Charcodote and Enterosgel with C.diff A.

1. Linear regression analysis of all the experimental isotherms was done using a linear adsorption isotherm (1) and linear forms of Langmuir and Freundlich two-parameter equations:

   Linear (Henry, H) equation: $q_e = K_H C_e$ (1)

   Langmuir equation (L): $$q_e = q_m \frac{K_L C_e}{(1+K_L C_e)}$$ (2)

   Linear form of Langmuir equation: $$\frac{1}{q_e} = \frac{1}{q_m K_L C_e} + \frac{1}{q_m}$$ (2a)

   For non-linear curve fitting this equation was used in the form of:

$$y = ax/(1 + bx), \qquad (2b)$$

where $a = q_m K_L$ and $b = K_L$

Freundlich equation (F): $$q_e = K_F C_e^{1/n} \qquad (3)$$

Linear form of Freundlich equation: $$lnq_e = lnK_F + {}^{1}/_{n}\, lnC_e \qquad (3a)$$

where q_e is amount adsorbed per amount of adsorbent at equilibrium (ng/g for TcdA, µg/g for TcdB and Stx-2B, and EU/g for endotoxin), c_e – final, or equilibrium concentration of the solution, q_m – maximum adsorption capacity for the complete monolayer coverage in the Langmuir model, K_L, K_F and 1/n are other parameters of these two-parameter equations.

The linear regression analysis performed with Excel showed poor correlation with either Langmuir or Freundlich equations, the highest $R^2 = 0.898$ being for Enterosgel – endotoxin, whereas other correlation coefficients are below 0.8. The results of linear curve fitting are not shown.

2. Non-linear regression analysis was done using Curve Expert Professional 2.6.5* and SigmaPlot 14 software using two-parameter Langmuir (2) and Freundlich (3) adsorption isotherm equations and three-parameter equations:

Toth equation (T):

$$\frac{q_e}{q_m} = \frac{K_T C_e}{[1+(K_T C_e^t)]^{1/t}} \qquad (4)$$

where q_m, K_T, and t are parameters [S2];

for curve fitting this equation was used in the form of:

$$y = ax/[(1 + bx^t)^{1/t}] \qquad (4a)$$

where $a = q_m K_T$, $b = K_T$

Langmuir equation in the form adapted for sigmoid (S-shape) isotherms (L-S):

$$q_e = q_m \frac{K_L C_e}{(1+K_L C_e + \frac{S}{C_e})} \qquad (5)$$

with S as the third parameter in the equation;

for curve fitting this equation was used in the form of:

$$y = x/(a + bx + c/x) \qquad (5a)$$

where $a = 1/(q_m K_L)$, $b = 1/q_m$ and $c = S/(q_m K_L)$ [S3],

and combined Langmuir-Freundlich (L-F) equation:

$$q_e = q_m \frac{K_L {C_e}^n}{(1+K_L {C_e}^n)} \qquad (6)$$

for curve fitting this equation was used in the form of:

$$y = x^n/(a + bx^n) \qquad (6a)$$

where $a = 1/(q_m K_L)$, $b = 1/q_m$

with parameters q_m, K_L and n similar to the two-parameter Langmuir (1) and Freundlich (2) equations [S4].

For obvious mathematical reasons, the point (0,0) could not be used in linear regression of the equations (2a) and (3a) as it required division by zero, but in non-linear regression this point was included. In L-S equation the point (0,0) could not be used either, and in the curve fitting the point (0.1: 0) was used instead. It should be noted that the equation parameters did not change significantly with or without (0,0) point.

References

S1. Lagergren, S. Zur theorie der sogenannten adsorption geloster stoffe, Kungliga Sevenska Vetenskapasakademiens, Handlingar, **24,** 1–39 (1898).

S2. Toth, J. State equations of the solid gas interface layer. Acta Chem. Acad. Hung. **69,** 311–317 (1971).

S3. Limousin, G., Gaudet, J.P., Charlet, L., Szenknect, S., Barthès, V., Krimissa, M. Sorption isotherms: A review on physical bases, modeling and measurement. Applied Geochemistry **22**(2), 249-275 (2007).

S4. Jaroniec, M., Derylo, A., Marczewski, A. The Langmuir-Freundlich equation in adsorption from dilute solutions on solids. Monatshefte fiir Chemie, **114**, 393--397 (1983).
